# Factors influencing urinary tract infection prevention and antibiotic stewardship in European nursing homes: an interview study with staff

**DOI:** 10.1007/s41999-025-01330-9

**Published:** 2025-10-21

**Authors:** Marie Theut, Alexandra Jønsson, Jette Nygaard Jensen, Valeria Antsupova, Malene Plejdrup Hansen, Carl Llor, Ana Garcia-Sangenis, Ana Moragas, Lina Jaruseviciene, Nina Sodja, Anna Kowalczyk, Andras Balint, Helena Glasova, Agapi Angelaki, Jesper Lykkegaard

**Affiliations:** 1 Research Unit for Antibiotic Stewardship and Implementation, Department of Clinical Microbiology, Copenhagen University Hospital - Herlev and Gentofte, Borgmester Ib Juuls Vej 52, 5., 2730 Herlev, Denmark; 2 Research Unit for General Practice, Department of Public Health, University of Southern Denmark, Odense, Denmark; 3 Department of People and Technology, Roskilde University, Roskilde, Denmark; 4 Department of Community Health, Arctic University of Norway, Tromsø, Norway; 5 Department of Clinical Microbiology, Copenhagen University Hospital – Herlev and Gentofte, Herlev, Denmark; 6 Center for General Practice at Aalborg University, Gistrup, Denmark; 7 Institut Català de la Salut, Barcelona, Spain; 8 CIBER de Enfermedades Infecciosas, Instituto de Salud Carlos III, Madrid, Spain; 9 Fundació Institut Universitari per a la Recerca a l’Atenció Primària de Salut Jordi Gol, Barcelona, Spain; 10 Jaume I Health Centre, University Rovira i Virgili, Tarragona, Spain; 11 Department of Family Medicine, Lithuanian University of Health Sciences, Kaunas, Lithuania; 12 Health Center Logatec, Logatec, Slovenia; 13 Department of Family Medicine, Faculty of Medicine, University of Ljubljana, Ljubljana, Slovenia; 14 Centre for Family and Community Medicine, The Faculty of Health Sciences, The Medical University of Lodz, Lodz, Poland; 15 Szeged Autumn Nursing Home, Szeged, Hungary; 16 Department of Clinical Pharmacology, Faculty of Medicine, Slovak Medical University, Bratislava, Slovakia; 17 Clinic of Social and Family Medicine, School of Medicine, University of Crete, Heraklion, Greece

**Keywords:** Nursing homes, Anti-bacterial agents, Hygiene, Urinary tract infections, Qualitative research

## Abstract

**Purpose:**

To explore the perceptions of European nursing home staff regarding factors influencing the prevention of urinary tract infections and antimicrobial stewardship.

**Methods:**

This qualitative descriptive study was conducted between July and September 2023. In each of eight European countries—Denmark, Greece, Hungary, Lithuania, Poland, Slovakia, Slovenia, and Spain—five semi-structured interviews were carried out with nursing home staff, resulting in a total of 40 interviews. All interviews were audio-recorded, transcribed verbatim, and analyzed using systematic text condensation.

**Results:**

Factors perceived to influence infection prevention and antibiotic stewardship were organized into five themes: (1) residents, including the respect for their autonomy, (2) relatives, including their demands for investigations and treatment, (3) physicians, including their attitudes towards antibiotics, (4) staff, including their challenging working conditions and lack of education, and (5) the nursing home environment in which these four actors navigate.

**Conclusion:**

Nursing home staff perceive multiple factors influencing infection prevention, including the challenge of balancing respect for residents’ autonomy with maintaining high hygiene standards. A key barrier to effective antimicrobial stewardship appears to be a lack of staff awareness regarding their crucial role in the process.

**Supplementary Information:**

The online version contains supplementary material available at 10.1007/s41999-025-01330-9.

## Key summary points

**Aim:**

This study aimed to explore nursing home staff’s perceptions of factors influencing infection prevention and antibiotic use in European nursing homes, focusing on urinary tract infections.

**Findings:**

Staff reported being in a challenging position, needing to balance respecting residents’ autonomy with maintaining proper hygiene. Regarding antibiotic stewardship, staff awareness of their crucial role was found to be limited.

**Message:**

Nursing home staff should be made more aware of their important role in the processes surrounding residents’ antibiotic use, and routines should balance residents’ hygiene needs with their autonomy.

**Supplementary Information:**

The online version contains supplementary material available at 10.1007/s41999-025-01330-9.

## Background

Antibiotic use is the main driver of antimicrobial resistance (AMR), which leads to increased morbidity, mortality, prolonged hospital stays, and consequently a higher economic burden on society. AMR is considered a serious threat to global health, and prudent use of antibiotics is therefore among the World Health Organization (WHO)’s top priorities [1, 2].

Nursing home residents are prescribed an excessive amount of antibiotics compared to older people living at home [3, 4], and the rate of antibiotic resistance is higher in nursing home settings than in the general community [5]. The HALT 4 survey, facilitated by the European Centre for Disease Prevention and Control (ECDC) found that, on any given day, 4.1% of all nursing home residents in Europe are treated with at least one antibiotic [6].

An estimated 40–75% of antibiotics used in nursing homes are assessed inappropriate, often due to unmet diagnostic criteria [3, 7–11]. Nearly half of the antibiotics target suspected urinary tract infections (UTIs), many of which could be avoided [3, 12]. Frequently, nursing home residents treated for UTIs lack specific symptoms such as urgency or painful urination and are instead treated for asymptomatic bacteriuria or non-specific symptoms. Asymptomatic bacteriuria affects 25–50% of female and 15–40% of male nursing home residents. It is a harmless condition that does not benefit from antibiotic treatment [12–14]. Non-specific symptoms such as cloudy or foul-smelling urine or confusion are sometimes interpreted as signs of UTI despite other possible causes, and current consensus advises against using antibiotics for these symptoms alone [15–19].

Key strategies to reduce antibiotic use include improving infection prevention and antimicrobial stewardship [20]. In nursing home settings, the residents’ personal hygiene as well as staff behavior and adherence to guidelines are important elements of infection prevention. Antimicrobial stewardship refers to coordinated efforts to optimize the use of antibiotics to treat infections effectively while minimizing the risk of overuse. In nursing homes, antimicrobial stewardship should involve optimizing diagnostics and the antibiotic prescription process, establishing guidelines for the management of ill residents, and providing education and training of staff [21]. Since infection prevention and antibiotic stewardship are closely linked, addressing them together is beneficial. However, nursing home studies exploring both strategies are scarce.

While it is the physicians who make the final decision to prescribe antibiotics, nursing home staff play a crucial role in the process by observing the residents, interpreting symptoms, and contacting and communicating with the physicians, who usually have limited direct contact with the residents [17, 19, 22–24]. A recent study showed that an intervention targeting nursing home staff’s communication skills and knowledge of UTIs halved antibiotic prescriptions without increasing hospitalizations or mortality, despite not involving physicians directly [23]. To properly equip the nursing home staff to fulfill this important role, a deeper understanding of the barriers and facilitators they face in infection prevention and antimicrobial stewardship is needed.

This study explores staff’s perceptions of factors influencing infection prevention and antimicrobial stewardship in nursing homes across eight European countries, focusing on urinary tract infections.

## Methods

The study is part of IMAGINE, an EU project focused on reducing antibiotic use in nursing homes through infection prevention and antimicrobial stewardship [25]. It includes 109 nursing homes across eight countries: Denmark, Greece, Hungary, Lithuania, Poland, Slovakia, Slovenia, and Spain. Table 1 provides an overview of the participating nursing homes and the diverse care settings in each country.

**Table 1:** Characteristics of the nursing homes in IMAGINE by country

	Denmark	Greece	Hungary	Lithuania	Poland	Slovakia	Slovenia	Spain
Number of nursing homes	15	9	18	15	12	10	15	15
Number of residents in each nursing home (mean)	76	49	85	52	120	92	161	75
Public ownership	Most	None	Most	Most	Most	Some	Most	Some
Qualified nursing care is available:								
During the day	All	All	Most	All	All	All	All	All
During the night	Some	All	Most	Some	Most	Most	Most	Some
During the weekend	Most	Some	Most	Some	All	Most	Most	Some
Medical activities in the nursing home:								
Are coordinated by a physician from inside or outside the nursing home	Most	All	All	Most	All	Most	All	All
Each resident has their own physician	Some	Some	Some	Some	Some	Some	None	None
Antibiotic use in the nursing home:								
A designated physician, either employed or not by the nursing home, prescribes antibiotics	Most	All	Most	Most	All	Most	Most	Most
Different physicians from outside the nursing home prescribe antibiotics	Most	None	Some	Some	Some	Some	Some	Some
Antibiotics can be provided without prescription	None	None	None	None	Some	Some	None	None
Proportion of residents who have their own room	All	Most	Some	Some	Some	Some	Some	Some
Proportion of resident’s rooms that have a hand wash sink	All	Most	Most	Most	All	Most	Most	Most

A staff member from each of five nursing homes per country was interviewed

### Study design

This qualitative study involved semi-structured interviews with nursing home staff from 40 of the 109 nursing homes included in IMAGINE—five per each of the eight participating countries. Selecting five participants per country aimed to capture diverse experiences while keeping the data manageable for analysis. The study is detailed in Annex I of the IMAGINE protocol [19]. The consolidated criteria for reporting qualitative research (COREQ) checklist was followed and is provided in Online Appendix I [26].

### Interviewers and workshop

In each participating country, a national interviewer was appointed from the research group. Prior to the interviews, all interviewers attended a 1-day workshop to ensure consistency in interview conduct across countries. The workshop also addressed the diverse nursing home settings in the eight countries and how to adapt interviews accordingly.

### Interview guide

Preceding the study, an online survey was conducted to gather basic knowledge of the context in which the nursing home staff work. The survey resulted in 286 responses from the participating nursing homes’ staff members. Based on these results and existing literature, an interview guide was developed with input from all the IMAGINE researchers during regular online meetings. MT pilot-tested the guide with staff from two Danish nursing homes, made adjustments, and presented it at the preparatory workshop. After further revision, the finalized interview guide (see Online Appendix II) covered three main topics: infection prevention procedures, antibiotic use procedures, and management of suspected UTIs. Developed in English, the guide was translated into native languages by the interviewers in each participating country.

### Data collection

As all interviewers were members of the IMAGINE research group, they were familiar with the nursing homes participating from their respective countries and selected five of them to take part in the qualitative study. Each selected nursing home then nominated one staff member to be interviewed. Informants were required to be actively involved in daily resident care, and efforts were made to select participants from different nursing homes and ensure diversity in education and roles.

Interviews were conducted between July and September 2023, in person at the informant’s nursing home when possible, or otherwise online. Each interview was scheduled to last 30–45 min. Basic demographic data, including education and role, were collected. Recorded interviews were pseudonymized using unique codes.

Interviews were conducted in the informants’ native languages, audio-recorded, and transcribed verbatim with identifying information removed. Transcripts were then translated into English by the interviewers. Coding was performed by MT, supervised by JNJ and experienced qualitative researchers AJ. Data saturation was reached, as the final interviews yielded no new codes [27]. Quotations included were carefully reviewed and refined by MT, in collaboration with country-specific researchers when needed, to ensure clarity and accuracy.

### Data analysis

The interviews were analyzed using NVivo following Malterud’s systematic text condensation method, involving four steps: (1) overall reading and theme identification, (2) coding meaning units, (3) condensing code content, and (4) synthesizing condensates into reconceptualized categories [28]. Regular online meetings were held within the international research team to discuss findings and resolve translation or interpretation issues.

After drafting the manuscript, ChatGPT was used for proofreading and refinement.

### Ethics

The study adhered to the IMAGINE project protocol, the Declaration of Helsinki, Good Clinical Practice principles, the EU General Data Protection Regulation (2016/679), the Human Research Act, and other relevant local regulations. The local interviewers informed the nursing home staff about the study, provided an information sheet and allowed time for questions. Staff who consented signed an informed consent form. Interviews were audio-recorded without personal identifiers and recordings were deleted immediately after transcription.

The study protocol received ethical approval in Spain, the coordinating country, from the Ethics Committee of IDIAP Jordi Gol, Institute of Research in Primary Health Care (ref. 23/080-P) on 12 July 2023.

## Results

For the interviews, the interviewer in each country predominantly selected nursing homes with which they had a good collaboration, that were positive about participating, and/or that were geographically nearby. The nursing home manager at each facility determined who would be interviewed.

Five interviews were conducted in each of the eight countries. In one of the interviews, two informants participated, resulting in a total of 41 individuals being interviewed: 25 nurses, 11 caregivers, assistants, or other types of healthcare staff, four physicians, and one psychologist. 32 of the interviews took place at the informant’s nursing home, while the remaining eight were conducted online. The research team is unaware of any refusals to participate.

At the beginning of the interviews, participants were asked about their tasks at the nursing home to ensure that only staff involved in the daily care of the residents were included. On that basis, interviews with physicians and the psychologist were only used as background. All quotations are from nurses and other healthcare staff, hereafter referred to as “informants” (*n* = 36).

**Fig. 1:**
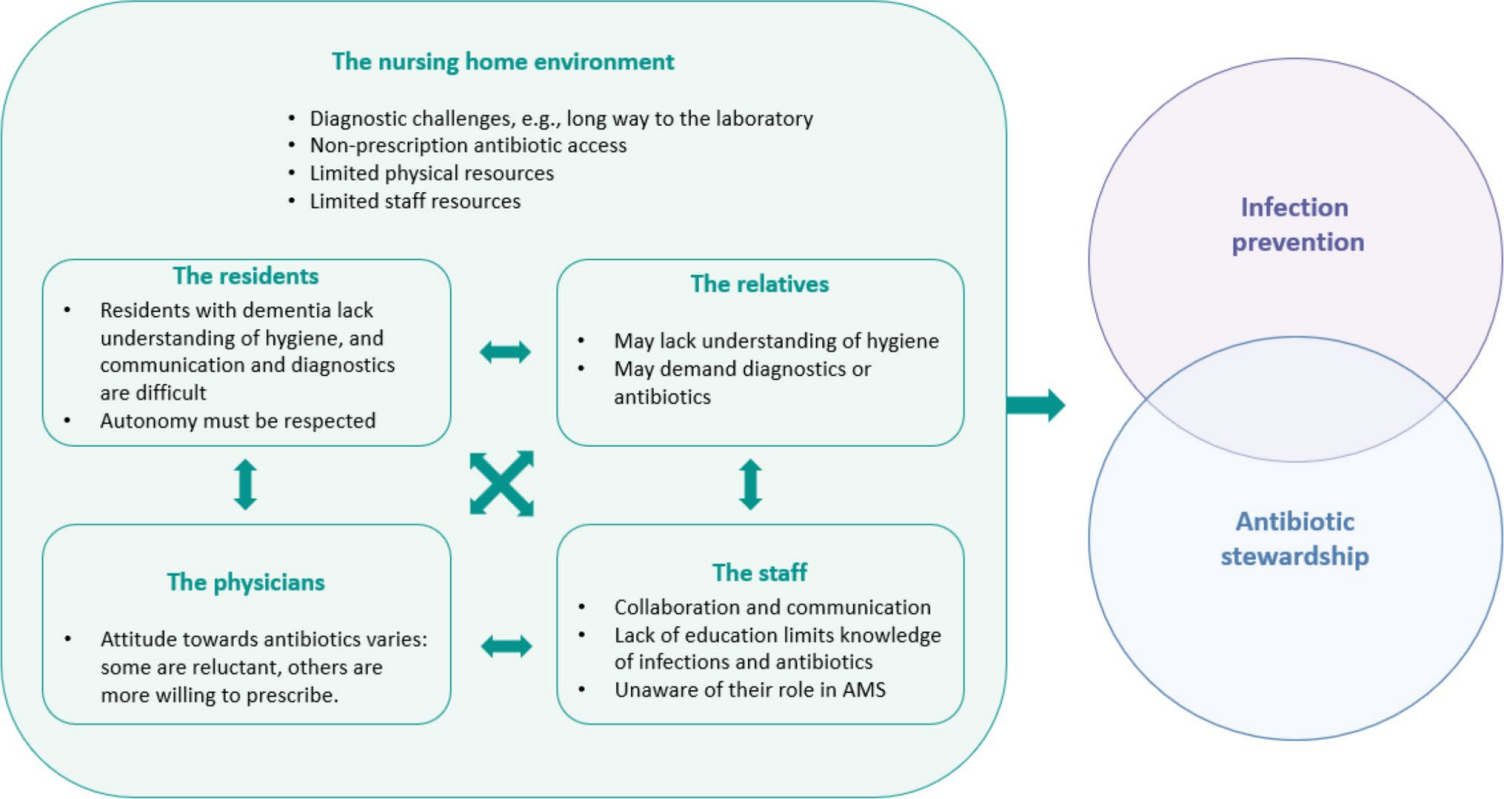
Factors influencing infection prevention and antibiotic stewardship in European nursing homes. The residents, relatives, physicians, and staff all operate within the nursing home environment

Several factors influencing infection prevention and antibiotic stewardship were identified and organized into five main themes related to residents, relatives, physicians, staff, and the nursing home environment (see Fig. 1). While each theme is presented separately, they are interconnected, with the nursing home environment (Theme 5) shaping the other four.

### Theme 1: The residents—and the respect for their autonomy

An important factor in infection prevention is residents maintaining good personal hygiene. While staff encourage this, they acknowledged it can sometimes be challenging:

*“There are 50 residents who walk around on their own and socialize with each other. They are very reluctant to wash or disinfect their hands”.*

SI5

Informants highlighted that residents with dementia present particular challenges. Cognitive impairment often hinders their understanding and adherence to infection prevention measures, such as proper hand hygiene. Additionally, assessing suspected infections is difficult, as these residents may struggle to describe their symptoms.

*“Certainly, there are greater difficulties with these residents because they cannot express what is happening to them. Other residents can tell me, for example, that during urination, they feel a burning sensation. However, in cases of dementia, I won’t receive such information. And, of course, I would act based on such information, but for those who cannot express themselves, you need to observe changes in behaviour, temperature, and urine.”*

GR1

As the quote above reveals, the staff must rely more on their observations than on the information they get from the residents.

*“Most demented residents have incontinency as well, so at changing diapers it can be seen or detected if there is any complaint that the resident could not tell us.”*

HU1

Furthermore, some residents with dementia resist procedures like temperature measurement or urine sampling, complicating accurate diagnosis. Informants also reported challenges with residents insisting on managing their personal hygiene despite limited capacity.

The fundamental issue, according to informants, is that nursing homes are residents’ homes, requiring staff to respect their autonomy more than in hospitals, where staff can enforce hygiene rules that patients must follow. This often places staff in a difficult position, balancing respect for autonomy with awareness of increased infection risk.

Informants also highlighted challenges to residents’ autonomy when managing contagious diseases or colonization with multidrug-resistant bacteria, such as methicillin-resistant *Staphylococcus aureus* (MRSA). Unlike hospitals, where isolation is standard to prevent transmission, nursing homes rarely implement isolation, increasing the staff’s responsibility to maintain strict hygiene.

### Theme 2: The relatives—sometimes being demanding

Informants described how collaboration with relatives and the relatives’ attitudes toward infection prevention and antibiotic use can affect resident care. While most relatives are cooperative, demonstrating trust in the staff and in hygiene precautions, some lack understanding of infection prevention, for example, by visiting the resident when they themselves are ill. Additionally, some relatives may disagree with staff decisions about the residents and may occasionally demand physician visits, hospital admission or discharge, diagnostic tests, or antibiotic treatment.

*“In general, relatives cooperate, but sometimes there are relatives who request antibiotics, or sometimes they request urine dipstick tests to be performed even without symptoms. We explain that if there are no symptoms, it is not indicated to perform the dipstick analysis. Some people understand it, but some people are more insistent, and in such cases, we refer them to the doctor to give them further explanations”.*

ES5

Sometimes, the staff members feel forced to contact a physician at the request of a relative, and the informants described how the physician’s decision is sometimes influenced by pressure from the relatives:

*“If there are no symptoms, treatment is not usually given, but sometimes there is a lot of pressure from the families, and they do not accept that antibiotics are not given. Currently, we have a lot of pressure from relatives who demand everything”.*

ES4

Occasionally, the physician prescribes antibiotics pushed by the relatives' demands:

“*It has occurred that antibiotics were prescribed for peace of mind”.*

SI4

The potential conflict with relatives thus poses the risk of overruling infection prevention and antibiotic stewardship.

### Theme 3: The physicians—and their attitude towards antibiotics

Informants emphasized that physicians play a key role in antibiotic use, as they are the ones to diagnose the residents and prescribe antibiotics. However, physicians often base their decisions on staff observations, since they are frequently absent from the nursing home.

Informants noted that physicians’ varying attitudes towards antibiotics influence antibiotic stewardship. Generally, informants reported that physicians tend to be somewhat reluctant to prescribe antibiotics and may adopt a watchful waiting approach depending on the resident’s condition. They often advise staff to monitor the resident for several days or encourage increased fluid intake to observe potential improvement. While most informants viewed this cautious approach as reasonable, some were more critical:

*“I feel like the physicians can be a bit, um, they’re a bit too hesitant. Because they want us to do a lot before prescribing antibiotics. And I do actually understand that, because, well, the symptoms may be due to hallucination or dehydration. Well, there could be many things. But when we get to the point where we contact the physician, then we HAVE already tried things. We HAVE given lots of fluids. Well, we’ve basically tried everything. Um, and it’s such a pity, because the residents get so terribly ill when they have an infection”.*

DK4

A few informants reported the opposite, describing physicians as eager to prescribe antibiotics even when staff questioned the appropriateness, and believed physicians should exercise more caution.

### Theme 4: The staff—key players in infection prevention, but challenged by a lack of training

The informants described staff as playing a significant role in infection prevention in the nursing homes. Collaboration and communication were reported as central aspects of the staff’s job and as key factors influencing infection prevention.

*“Probably the best thing is that our team is very strong, and we know what each person is doing at any given moment. There’s a very, very strong mutual connection among all of us”.*

LT2

A major challenge reported by informants is high staff turnover and extensive use of temporary workers. Many staff members lack healthcare education, resulting in insufficient knowledge of infection prevention and antibiotic use. This knowledge gap includes difficulty recognizing signs and symptoms that should prompt infection suspicion and physician contact. Notably, there is a poor understanding of asymptomatic bacteriuria as a harmless condition, which can lead to overtreatment.

“*When we have new employees, I have to constantly check them. I see this as one of the hardest problems. For example, during hygiene with the resident, they throw bed linens on the floor. I say repeatedly that it must not end there, that it is a risk of transmission of infections, as well as placing it on the other bed or on another piece of furniture”.*

SK2

Informants clearly recognize the staff’s crucial role in infection prevention, which is emphasized throughout the interviews. In contrast, staff involvement in antibiotic stewardship receives little attention, and informants generally seem unaware of its importance. Most attribute antibiotic stewardship primarily to physicians, as they prescribe the antibiotics, though some acknowledge that staff also play a role since the prescribing process begins with them.

### Theme 5: The nursing home environment—diagnostic challenges and lack of resources

Informants identified several nursing home environmental factors affecting infection prevention and antibiotic stewardship. Diagnostic options are limited compared to hospitals; for example, sending urine samples for laboratory culture is cumbersome and slow. As a result, residents are often prescribed antibiotics empirically, without confirmed diagnoses.

*“If the CRP [c-reactive protein blood test]is 35 and above, then Biseptol [sulfamethoxazole-trimethoprim] is given empirically first, for 3 days. If the symptoms do not decrease and the resident indicates pain, the temperature rises and CRP increases, if it progresses and the condition worsens, Ciprinol [ciprofloxacin] is used”.*

SK1

Additionally, informants noted that in some countries and situations, residents may receive antibiotics without physician involvement, directly impacting antibiotic use.

*“The decision is usually made by the physician, but there are also situations where we decide for ourselves that we need to give Cipronex [ciprofloxacin] or other antibiotics.”*

PL1

Another key factor influencing infection prevention and antibiotic stewardship is resource scarcity, including both physical supplies and staffing. Physical resource limitations were reported mainly in some countries—for example, restrictions on the number of diapers and gloves per resident—while in others, this was less of a concern.

“*The resources are limited, meaning that we cannot ask our boss for 10 boxes of gloves a day, as is the case in hospitals”.*

GR4

Furthermore, staff at nursing homes where residents share rooms identified this as a particular infection prevention challenge. Additionally, the absence of sinks in some residents’ rooms complicates handwashing, increasing infection risk (see Table 1).

Several informants emphasized busyness as a key issue, citing excessive tasks and insufficient time. This is linked to overall staff shortages, particularly of permanent, skilled personnel. High staff turnover and reliance on untrained or temporary workers unfamiliar with nursing home routines were also noted.

Some informants reported that being rushed undermines infection prevention. For example, personal protective equipment is sometimes omitted, and hand disinfection may be rushed or incomplete. Staff also lack time to encourage residents to drink—despite knowing its importance in preventing UTIs—or to closely observe residents when infections are suspected. Additionally, busyness limits time for properly training new and temporary staff, increasing pressure on permanent staff and creating a vicious cycle. Thus, several factors in the nursing home environment influence infection prevention and antibiotic stewardship, either directly or indirectly through residents, relatives, physicians, or staff.

## Discussion

Our analysis identified multiple factors influencing infection prevention and antibiotic stewardship in nursing homes. We found that residents, relatives, physicians, and staff all play significant roles shaped by the nursing home environment.

Consistent with prior research, we found that relatives can influence antibiotic use by requesting unnecessary diagnostics or antibiotics, to which physicians may sometimes acquiesce to appease them [17–19, 22, 29].

We also found that staff face suboptimal working conditions, including high workloads, staff turnover, insufficient training, and frequent reliance on temporary workers —factors previously linked to increased antibiotic prescribing [17, 18, 24, 30]. Additionally, previously identified factors influencing antibiotic use in nursing home settings include aspects related to the nursing home environment. Diagnostic challenges in these settings are well-documented [17, 31] and align with our findings. Obtaining and sending samples to laboratories is more cumbersome and time-consuming than in hospitals, and the sampling process is further complicated by the cognitive impairments of many nursing home residents. Additionally, in some countries, antibiotics may be administered without a physician’s prescription, a practice known to affect antibiotic stewardship [18].

Our study offers new insights into factors influencing infection prevention in nursing homes. The findings emphasize the balance of respecting residents’ autonomy when it conflicts with infection prevention standards [32]. Professionals play an ambivalent role facilitating and hindering autonomy, as they balance protective and restrictive hygiene measures. In our results, staff reported challenges when residents refuse assistance with personal hygiene or, in cases of likely contagious conditions, insisted on socializing with others. These situations place staff in the difficult position of balancing two competing priorities: respecting residents' autonomy while ensuring proper hygiene to prevent infections in both the affected resident and the broader community. This is especially challenging with residents suffering from cognitive impairment, including dementia. To our knowledge, these dilemmas have not been explored in previous research, except in studies examining the ethical challenges of isolating nursing home residents during the COVID-19 pandemic [33].

Another novel finding of this study is that while healthcare staff fully recognize their important role in infection prevention, they only partially acknowledge their role in antibiotic stewardship. Many perceive antibiotic stewardship as beyond their control, attributing it solely to the physicians, since they are the ones who prescribe antibiotics. However, healthcare staff play a crucial role since the process of detecting potential infections starts with them. Being in daily contact with the residents, they notice when a resident’s behavior deviates from the norm, and if they find it necessary, they consult a physician. Based on their observations, the physician determines the further procedure, including the prescription of antibiotics. Therefore, contacting the physician only when necessary and conveying relevant and accurate information is essential for antibiotic stewardship. This responsibility is challenged by the high number of temporary and untrained staff, who often lack essential knowledge of infections and antibiotics. Limited time for training further exacerbates this issue, underscoring the need for comprehensive education programs to empower all staff in antibiotic stewardship.

Regarding UTIs, diagnosing nursing home residents is particularly challenging due to the high prevalence of asymptomatic bacteriuria and nonspecific signs and symptoms, with no reliable algorithm or test available [34]. Our results indicate that many nursing home staff are unaware that asymptomatic bacteriuria is common and harmless in this population and does not require antibiotic treatment. They also often struggle to distinguish true UTI symptoms from nonspecific signs, leading to unnecessary physician consultations driven by fear of missing an infection—situations that better knowledge could prevent. Staff often do not recognize that their decision to contact a physician directly affects antibiotic prescribing, a lack of awareness that may contribute to overdiagnosis and overtreatment [35, 36]. Our recent study showed that more than one-third of nursing home residents treated for suspected UTI exhibited only nonspecific symptoms, such as poor general condition and changes in urine appearance, while specific urinary symptoms, such as incontinence and dysuria were less common [11]. Studies have shown that although it is the physicians who prescribe antibiotics, enhancing staff knowledge about infections and antibiotics can reduce antibiotic use in nursing home settings [23]. A lack of understanding about antibiotic drawbacks may also explain why staff often view physicians as too reluctant to prescribe, as reflected in our findings.

In conclusion, enhancing healthcare staff’s knowledge of infections and antimicrobials—and especially their understanding of their important role in antibiotic stewardship—is essential to reduce antimicrobial use.

No participants mentioned language barriers, which we would have expected to be challenging since immigration is considerable in many European countries. Current immigrants may work in nursing homes and aged immigrants may move into nursing homes. Maybe this is less in the participating countries, or we failed to address this via the interview guide. It is unlikely to have been considered by the participants as a major barrier affecting UTI care.

The main strength of this study is its inclusion of diverse countries, cultures, and nursing home settings, enabling the findings to inform research and interventions across varied cross-cultural contexts. The nursing homes included in the study vary in several respects, including available resources, staff-to-resident ratios, and the proportion of residents with dementia or those using wheelchairs—all factors that influence the working conditions and challenges faced by staff. By including a broad range of nursing homes, we were able to capture a wide spectrum of experiences and thereby gaining a more comprehensive understanding of prevention and antimicrobial stewardship regarding UTIs in nursing homes.

The only inclusion criterion for interview participants was that they had to be involved in the daily care of the residents, resulting in a mix of nurses and various care staff among those interviewed. From the interviews, it became clear that professional roles and responsibilities vary between countries and even between individual nursing homes, so tasks performed by nurses in one country may be primarily carried out by other professional groups in another—and vice versa. We consider it a strength of the study that the views of most possible types of involved professionals are represented, as this increases the completeness of information. Since the professions and roles are not identical across countries, we have not tried to report profession-specific views.

Conducting interviews in the native language by local interviewers is both a strength and a limitation. The use of the native language allowed informants to feel comfortable and speak freely, and local interviewers likely had a better understanding of the cultural context compared to outside expert interviewers. However, the local interviewers conducted and transcribed the interviews in the native language and then translated them into English. The data were subsequently analyzed by the Danish researcher MT, a medical doctor primarily familiar with the Danish nursing home setting. Inevitably, some nuances of the informants' statements may have been lost or misunderstood in this process. To mitigate this impact, MT sought clarification from the interviewers whenever there was doubt about the meaning.

Within the research team, AM, LJ, AK, AB, HG, JL, and AA had prior qualitative research experience, while AA, AB, and NS were familiar with nursing homes in their countries. Differences in interviewing experience and nursing home familiarity posed a limitation, potentially influencing the interviews. To enhance consistency, all interviewers attended a qualitative workshop and used the same interview guide.

All interviewers were involved in the IMAGINE study, which aims to reduce infections and unnecessary antibiotic use in nursing homes. Consequently, they had prior knowledge of these issues but may also hold a bias that antibiotic use is excessive. This may have influenced both their interviewing approach and the responses obtained.

A further limitation of the study is that we did not obtain demographic information such as the sex and age of the informants. This decision was made to protect their confidentiality.

One of our main findings was that the residents’ autonomy posed a challenge to infection prevention. In the interviews, staff were not directly asked about autonomy, but some mentioned it spontaneously. Since this emerged as a key finding, it is a limitation that not all staff were asked about it, as those who did not bring it up may have had different experiences than those expressed here.

Additionally, the finding that nursing home staff are not fully aware of their crucial role in AMS was not explicitly stated by the staff themselves but rather a conclusion drawn during our analysis of the interviews.

It is important to note that while our study identified key themes regarding infection prevention and antimicrobial stewardship, it cannot be used to compare the included countries with each other, since only five participants were included from each country. A quantitative study would be more suitable for such a comparison.

Our aim was to provide in-depth insights into the experiences and perspectives within the specific settings studied. We believe that the data collected possess sufficient information power to thoroughly address our research question.

The facilitators and barriers identified may vary across settings and countries, warranting further exploration through surveys. Future research should examine how nursing home conditions, resources, and staff roles relate to these factors. Our study provides a foundation for such work and for interventions to improve infection prevention and antibiotic stewardship.

The findings highlight important directions for future research. It is essential to design infection prevention protocols that balance professional standards with residents’ autonomy and preferences. Further studies should examine the long-term effects of autonomy on health outcomes and quality of life in nursing homes. Additionally, interdisciplinary approaches are needed to develop strategies that incorporate residents’ autonomy into infection prevention without compromising safety.

The study findings can guide efforts to prevent infections and reduce unnecessary antibiotic use in nursing homes. Interventions should address the identified facilitators and barriers. Within the IMAGINE project, educational tools will target key barriers [20]. Effective antibiotic stewardship interventions must recognize the crucial role of staff and their working conditions, including the challenges of caring for frail older residents and balancing clinical practice with respecting residents’ privacy and autonomy—acting simultaneously as rule-keepers, caretakers, and guests in residents’ homes.

## Conclusion

This study highlights multiple factors influencing infection prevention and antibiotic stewardship in nursing homes, involving staff, physicians, residents, and relatives. Key findings include the challenge that respecting residents’ autonomy poses to infection prevention, as well as the staff’s limited awareness of their vital role in antibiotic stewardship. Targeted interventions are needed to enhance staff knowledge of infections, symptoms, antibiotics, and their critical role in AMS.

## Supplementary Information

Below is the link to the electronic supplementary material.

Supplementary file1 (DOCX 29 KB)

Supplementary file2 (DOCX 136 KB)

## Data Availability

The data that support the findings of this study are not openly available due to reasons of sensitivity and are available from the corresponding author upon reasonable request.
