# Supplementary material for: Factors influencing urinary tract infection prevention and antibiotic stewardship in European nursing homes: an interview study with staff
Source: Eur Geriatr Med. 2025 Oct 21;16(6):2293–303. doi: 10.1007/s41999-025-01330-9 (PMC12743712; doi:10.1007/s41999-025-01330-9)
Supplement: Supplementary file 1 — Supplementary file1 (DOCX 29 KB) [file 41999_2025_1330_MOESM1_ESM.docx]

**Appendix I: Consolidated criteria for reporting qualitative studies (COREQ): 32-item checklist** (1)

| **No. Item** | **Guide questions/description** | **Details reported here or in the main text of the paper** |
| --- | --- | --- |
| **Domain 1: Research team and reﬂexivity** | | |
| *Personal Characteristics* | | |
| 1. Interviewer/facilitator | Which author/s conducted the interview or focus group? | Marie Theut (MT), Denmark,  Ana Moragas (AM), Spain,  Lina Jaruseviciene (LJ), Lithuania,  Nina Sodja (NS), Slovenia,  Anna Kowalczyk (AK), Poland,  András Bálint (AB), Hungary,  Helena Glasova (HG), Slovakia,  Agapi Angelaki (AA), Greece |
| 2. Credentials | What were the researcher’s credentials? E.g. PhD, MD | All authors have a background in research and/or medical practice, most often in the field of general practice:  MT is a MD and PhD student,  AM is a family physician,  LJ is a family physician and PhD,  NS is a family physician and PhD,  AK has a Master of Public Health,  AB is Dr Med,  HG is a MD and PhD,  AA has a Master of Public Health |
| 3. Occupation | What was their occupation at the time of the study? | Researcher and/or physician |
| 4. Gender | Was the researcher male or female? | The research team has both male and female researchers. |
| 5. Experience and training | What experience or training did the researcher have? | Within the research team, AM, LJ, AK, AB, HG, and AA have previous qualitative research experience.  All interviewers received internal training in qualitative research during the workshop described in the paper. |
| *Relationship with participants* | | |
| 6. Relationship established | Was a relationship established prior to study commencement? | No |
| 7. Participant knowledge of the interviewer | What did the participants know about the researcher? e.g. personal goals, reasons for doing the research | Prior to each interview, the participants were shortly informed about the purpose of the IMAGINE project and the interview study.  The interviewer presented him-/herself by name and profession. |
| 8. Interviewer characteristics | What characteristics were reported about the interviewer/facilitator? e.g. Bias, assumptions, reasons and interests in the research topic | All the interviewers were part of the IMAGINE project research team and were therefore biased by the assumption that too many infections occur in nursing homes, and that too many antibiotics are used. |

| **Domain 2: study design** | | |
| --- | --- | --- |
| *Theoretical framework* | | |
| 9. Methodological orientation and Theory | What methodological orientation was stated to underpin the study? e.g. grounded theory, discourse analysis, ethnography, phenomenology, content analysis | Data was analyzed using the method systematic text condensation, described by Malterud et al. This method is inspired by Giorgi’s psychological phenomenological analysis (2). |
| *Participant selection* | | |
| 10. Sampling | How were participants selected? e.g. purposive, convenience, consecutive, snowball | In each country, the interviewer contacted up to five nursing homes, located relatively nearby. The nursing home staff chose one or more staff members involved in the daily care of residents to participate. |
| 11. Method of approach | How were participants approached? e.g. face-to-face, telephone, mail, email | Face-to-face |
| 12. Sample size | How many participants were in the study? | 41 |
| 13. Non-participation | How many people refused to participate or dropped out? Reasons? | As participants were recruited via nursing staff, no exact numbers or reasons for refusing to participate are available.  Of the 41 interviewed staff members, no one dropped out afterwards. |
| *Setting* | | |
| 14. Setting of data collection | Where was the data collected? e.g. home, clinic, workplace | 22 interviews took place in the nursing home, the remaining eight were conducted online. |
| 15. Presence of non-participants | Was anyone else present besides the participants and researchers? | No |
| 16. Description of sample | What are the important characteristics of the sample? e.g. demographic data, date | Nursing home staff involved in the daily care of the residents. |
| *Data collection* | | |
| 17. Interview guide | Were questions, prompts, guides provided by the authors? Was it pilot tested? | The interview guide was developed by MT in collaboration with all other interviewers.  MT pilot tested the interview guide. |
| 18. Repeat interviews | Were repeat interviews carried out? If yes, how many? | No |
| 19. Audio/visual recording | Did the research use audio or visual recording to collect the data? | All interviews were audio-recorded and transcribed verbatim. |
| 20. Field notes | Were ﬁeld notes made during and/or after the interview or focus group? | No |
| 21. Duration | What was the duration of the interviews or focus group? | The interviews lasted around 30 – 60 minutes |
| 22. Data saturation | Was data saturation discussed? | Yes |
| 23. Transcripts returned | Were transcripts returned to participants for comment and/or correction? | No |
| **Domain 3: analysis and ﬁndings** | | |
| *Data analysis* | | |
| 24. Number of data coders | How many data coders coded the data? | 1 (MT) supervised by AJ and JN |
| 25. Description of the coding tree | Did authors provide a description of the coding tree? | Yes, see Figure 1. |
| 26. Derivation of themes | Were themes identiﬁed in advance or derived from the data? | Derived from the data. |
| 27. Software | What software, if applicable, was used to manage the data? | NVivo software was used. |
| 28. Participant checking | Did participants provide feedback on the ﬁndings? | No |
| *Reporting* |  |  |
| 29. Quotations presented | Were participant quotations presented to illustrate the themes/ﬁndings? Was each quotation identiﬁed? e.g. participant number | Yes |
| 30. Data and ﬁndings consistent | Was there consistency between the data presented and the ﬁndings? | Yes |
| 31. Clarity of major themes | Were major themes clearly presented in the ﬁndings? | Yes |
| 32. Clarity of minor themes | Is there a description of diverse cases or discussion of minor themes? | Yes |

1. Tong A, Sainsbury P, Craig J. Consolidated criteria for reporting qualitative research (COREQ): a 32-item checklist for interviews and focus groups. Int J Qual Health Care. 2007 Sep 16;19(6):349–57.

2. Malterud K. Systematic text condensation: a strategy for qualitative analysis. Scand J Public Health. 2012 Dec;40(8):795–805.
