# Supplementary material for: Factors influencing urinary tract infection prevention and antibiotic stewardship in European nursing homes: an interview study with staff
Source: Eur Geriatr Med. 2025 Oct 21;16(6):2293–303. doi: 10.1007/s41999-025-01330-9 (PMC12743712; doi:10.1007/s41999-025-01330-9)
Supplement: Supplementary file 2 — Supplementary file2 (DOCX 136 KB) [file 41999_2025_1330_MOESM2_ESM.docx]

Interview guide

Instruction: **Bold-printed questions** are essential questions to be asked in the interview, the remaining questions can be used to raise follow-up questions.

Please see the “Checklist” and the “Tips and tricks for interviewing” before starting the interview.

| **Introduction**  (Feel free to use your own words, just make sure that all formalities are mentioned) | Thank you for participating.  My name is…. and I’m a…  The purpose of this interview is to obtain knowledge about the routines and challenges that you face in your work at the nursing home regarding infection prevention and the use of antibiotics.  The interview is expected to last approximately 30-45 minutes.  The interview will be recorded and transcribed, and the recordings will be deleted afterwards.  Five nursing home staff members in each of the eight countries participating in the IMAGINE project will be interviewed. It will not be possible to identify your interview from the others. Your name or the name of your nursing home will not appear anywhere in the publication.  The results of the 40 interviews will be published and will help us develop the intervention material to be used in IMAGINE. |
| --- | --- |
| Background of the participant | Can you tell me a little bit about yourself and your background?  What is your role at the nursing home?  How long have you been working here?  Do you have any special involvement in infection prevention or the use of antibiotics in your nursing home? |

| **Theme 1: General procedures and infection prevention**  **(What facilitates infection prevention and which are the barriers?)** | **Interview questions** |
| --- | --- |
| Opening question  Infection prevention  Hand hygiene  Procedure when suspecting an infection  Relatives | **Which tasks have you done/are you going to do today?**  Have/will any of your tasks be related to preventing infections? (Examples)  **Which daily tasks and routines do you have in your nursing home to prevent infections?**  Do you experience any challenges with these?  To what extent do you feel encouraged and supported by your workplace to prioritize infection prevention and incorporate infection preventive actions into your daily work?  **Where do you receive information or guidelines on how to perform infection prevention in your nursing home?**  **In your opinion, do you have many infections in your nursing home?**  What do you think might be the reason for this?  **Regarding hand hygiene, which routines do you have?**  Are there any situations in which it is difficult to perform hand hygiene? Which and why?  **Now I would like you to think of a resident that you suspect might have any kind of infection.**  **What do you do?** Who do you contact? What kind of information do you give the nurse/doctor/colleague? Do you experience any difficulties in the process? Which? Are there any difficulties to your colleagues, do you think?  **Is the procedure the same on weekdays and weekends?** (Describe)  **In general, how would you describe the collaboration with the relatives of the residents when it comes to infections?**  Have you experienced relatives that demanded antibiotics for the resident?  **In your experience, how much do the relatives know about infection prevention?**  How much and what do they do to prevent transmissions of infections at the nursing home? |

| **Theme 2: Antibiotic use**  **(what facilitates antibiotic stewardship and which are the barriers?)** | **Interview questions** |
| --- | --- |
| Use of antibiotics  Resistance | **When do your residents get antibiotics?**  **Who makes the decision of prescribing them?**  **Who is responsible for administering the prescribed antibiotics to the residents?**  **For which conditions have you experienced positive effects of antibiotics among the residents**?  (Examples)  **Have you experienced any disadvantages of using antibiotics?**  (Examples)  **What do you think about prophylactic antibiotic therapies for preventing urinary tract infections?**  Do you think they are useful? Do you see any disadvantages?  **Do you have any knowledge of antibiotic resistance in your nursing home?**  **Would you consider it a problem in your nursing home? Why (not)?**  **Do you have any suggestions on how to reduce the use of antibiotics in your nursing home?** |
| **Theme 3: Urinary tract infections** | **Interview questions** |
| Prevention  Indwelling urinary catheters  Challenging residents | **In the following questions, I would like to focus on urinary tract infections (UTIs).**  **Do you consider UTIs to be a problem in your nursing home? Why (not)?**  **To what extent do you believe it is possible to prevent UTIs?**  **What do you do in your nursing home to prevent UTIs?**  Are there any barriers that make it difficult for you to integrate these actions? Which and why?  What facilitates the prevention of UTIs in your nursing home?  **For residents with indwelling urinary catheters, what do you do to prevent UTIs?**  Are there any barriers that make it difficult for you to integrate these actions? Which and why?  What facilitates the prevention of UTIs in these residents?  **Which signs and symptoms lead you to suspect a UTI in a resident?**  **When you suspect a UTI in a resident, what actions do you take?**  What signs and symptoms prompt you to perform a urine dipstick test?  When do you decide to collect a urine sample for microbiological analysis?  **Let’s consider a scenario where the urine sample shows bacteria in the urine, but the resident has no symptoms from the urinary tract. What are your thoughts on this situation?**  How certain are you that the resident should be treated with antibiotics?  What makes you certain/uncertain?  **How do you manage residents with whom cooperation is challenging, such as those with dementia, when you suspect they might have a UTI?** |
| **Finish** | **Do you have any personal suggestions for an intervention or any materials that could help optimize the infection prevention and the use of antibiotics in your nursing home?**  **Is there anything else you think I should know about infection prevention or the use of antibiotics at the nursing home?**  Do you have anything to add?  Once again, thank you for your participation. |
